# Dichotomy in Fatal Outcomes in a Large Cohort of People Living with HTLV-1 in São Paulo, Brazil

**DOI:** 10.3390/pathogens9010025

**Published:** 2019-12-26

**Authors:** Rosa Maria N. Marcusso, Johan Van Weyenbergh, João Victor Luisi de Moura, Flávia Esper Dahy, Aline de Moura Brasil Matos, Michel E. J. Haziot, Jose E. Vidal, Luiz Augusto M. Fonseca, Jerusa Smid, Tatiane Assone, Jorge Casseb, Augusto César Penalva de Oliveira

**Affiliations:** 1 Institute of Infectious Diseases “Emilio Ribas” (IIER) of São Paulo, São Paulo 01246-000, Brazil; jvluisimoura@gmail.com (J.V.L.d.M.); esper.flavia@gmail.com (F.E.D.); michelhaziot@gmail.com (M.E.J.H.); josevibe@gmail.com (J.E.V.); jerusa.smid@emilioribas.sp.gov.br (J.S.); 2 Department of Microbiology, Immunology and Transplantation, Rega Institute for Medical Research, KU Leuven, 3000 Leuven, Belgium; johan.vanweyenbergh@kuleuven.be; 3 Institute of Tropical Medicine of São Paulo, São Paulo 05403-000, Brazil; alinembmatos@hotmail.com (A.d.M.B.M.); luizaugusto.marcondes@gmail.com (L.A.M.F.); jcasseb10@gmail.com (J.C.); 4 Laboratory of Dermatology and Immunodeficiencies, Department of Dermatology, Medical School, University of São Paulo Brazil, São Paulo 05403-000, Brazil

**Keywords:** HTLV-1, mortality, HAM/TSP, Brazil

## Abstract

**Background:** Despite its relatively low incidence of associated diseases, Human T-cell Leukemia Virus-1 (HTLV-1) infection was reported to carry a significant risk of mortality in several endemic areas. HTLV-1-associated diseases, adult T-cell leukemia/lymphoma (ATLL) and HTLV-1-associated myelopathy/tropical spastic paraperesis (HAM/TSP), as well as frequent coinfections with human immunodeficiency virus (HIV), hepatitis C virus (HCV), and *Strongyloides stercoralis* were associated to increased morbidity and mortality of HTLV-1 infection. **Objective:** To determine the mortality rate and its associated variables from an open cohort started in July 1997 at the HTLV Clinic, Emilio Ribas Institute (IIER), a major infectious disease hospital in São Paulo, Brazil. **Methods:** Since inception up to September 2018, we admitted 727 HTLV-1-infected individuals, with a rate of 30–50 new admissions per year. All patient data, including clinical and laboratory data, were regularly updated throughout the 21-year period, using a dedicated REDCap database. The Ethical Board of IIER approved the protocol. **Results:** During 21 years of clinical care to people living with HTLV-1 in the São Paulo region, we recruited 479 asymptomatic HTLV-1-infected individuals and 248 HAM/TSP patients, of which 632 remained under active follow-up. During a total of 3800 person-years of follow-up (maximum follow-up 21.5 years, mean follow-up 6.0 years), 27 individuals died (median age of 51.5 years), of which 12 were asymptomatic, one ATLL patient and 14 HAM/TSP patients. HAM/TSP diagnosis (but neither age nor gender) was a significant predictor of increased mortality by univariate and multivariate (hazard ratio (HR) 5.03, 95% CI [1.96–12.91], *p* = 0.001) Cox regression models. Coinfection with HIV/HCV was an independent predictor of increased mortality (HR 15.08; 95% CI [5.50–41.32]; *p* < 0.001), with AIDS-related infections as a more frequent cause of death in asymptomatics (6/13; *p* = 0.033). HIV/HCV-negative fatal HAM/TSP cases were all female, with urinary tract infection and decubitus ulcer-associated sepsis as the main cause of death (8/14, *p* = 0.002). **Conclusions:** All-cause mortality among people living with HTLV-1 in São Paulo differs between asymptomatic (2.9%) and HAM/TSP patients (7.3%), independent of age and gender. We observe a dichotomy in fatal cases, with HAM/TSP and HIV/HCV coinfection as independent risk factors for death. Our findings reveal an urgent need for public health actions, as the major causes of death, infections secondary to decubitus ulcers, and immune deficiency syndrome (AIDS)-related infections, can be targeted by preventive measures.

## 1. Background

Human T-cell Leukemia Virus-1 (HTLV-1), the first human retrovirus discovered, is the causative agent of adult T-cell leukemia/lymphoma (ATLL) and HTLV-1-associated myelopathy/tropical spastic paraperesis (HAM/TSP) [1,2], with approximately 5–10 million people infected worldwide, almost one million of them in Brazil [3]. The number of infected people may be higher, considering that only 2/3 of the world has been mapped for HTLV-1 infection [3]. After the adoption of mandatory national blood screening tests in 1993 and the observed behavioral change from intravenous to inhalation drug abuse, parental exposure to HTLV-1/2 dropped [4,5,6]. However, our group has documented rates of 40% of sexual transmission [6] and of 13% of vertical transmission [7,8].

The incidence of ATLL and HAM/TSP varies from 0.5% to 10% among HTLV-1 infected subjects, increasing morbidity and mortality rates [9] Additionally, treatment of coinfections, such as HIV and HCV, can be delayed due to HTLV-1 promoting an inefficacious increase in CD4+ cells, leading to a delay in antiretroviral therapy (ART) initiation and, possibly, inefficacious HCV clearance [10,11,12,13]. In endemic countries, parasitic coinfection with *Strongyloides stercoralis* also increases morbidity and mortality [12].

Previous studies in Japan, Australia, West-Africa, and the US observed variable mortality risks among HTLV-1-infected cohorts [14,15,16,17,18,19]. Despite the large variability among these populations, all studies reported that HTLV-1 by itself increased the risk for increased mortality [14,15,16,17,18,19], although confounding factors should be taken into account [18]. A recent systematic review and meta-analysis confirmed a significantly increased risk (RR 1.57) for mortality in people living with HTLV-1 [20]. Here, we report data spanning more than two decades of follow-up of a cohort of people living with HTLV-1 from a single tertiary Brazilian center, regarding mortality and related risk factors.

## 2. Methods

### 2.1. Population

We retrospectively reviewed medical records from the HTLV-1 cohort from the outpatient clinic of “Instituto de Infectologia Emílio Ribas” (IIER), Sao Paulo city, Brazil. IIER is a public tertiary infectious disease reference hospital with an HTLV-1 outpatient service since 1997, with new patients added at a rate of approximately 30–50 patients/year [21]. The included patients were at least 18 years old and had to have tested positive to HTLV-1 through third generation EIA (Murex I/II, Abbott Murex Diagnostic, Dartford, UK), confirmed by Western-blot (Soi Prasarnjai, Yannaw, Thailand) and/or polymerase chain reaction (PCR) [22]. Patients with incomplete data and/or lost to follow-up were excluded for this analysis.

### 2.2. Clinical Follow-Up

All individuals were evaluated by neurologist/infectious diseases specialists at least once in the 12 months previous to the start of data collection for this study (September 2018). Clinical variables concerning neurological, dermatological, ophthalmological, rheumatologic, urological, and buccal aspects were stored in a previously validated electronic database using RedCap^®^ [23]. People living with HTLV-1 were classified at recruitment as asymptomatic or HAM/TSP, according to the diagnostic criteria proposed by Castro-Costa et al. [24]. HAM/TSP severity was assessed by Osame motor disability scale (OMDS) [25]. The time frame for data collection was from July 1997 to December 2018.

### 2.3. Statistical Analysis

Statistical analysis was conducted using Mann–Whitney test for nonparametric data, and Chi-square or Fisher exact test for proportions. Univariate and multivariate Cox proportional hazard analysis was performed to identify independent variables associated with the risk of death, with Wald test for hazard ratio (HR) beta coefficient. For survival analysis, the Kaplan–Meier method was used to compare the probability of survival and the median survival time, and the log-rank test to compare survival curves. Death rate was expressed as 100 person-years; time was measured since the start of follow-up until death. Survival analysis was performed using SPSS^®^ 21 (Statistical Package for the Social Sciences-21. Statistical Software. IBM, NY, USA).

### 2.4. Ethical Issues

The study was approved by IIER’s ethical board under the protocol 86806218.9.0000.0061. Signed informed consent was obtained from all participants prior to study inclusion. This study follows the principles of the Declaration of Helsinki.

## 3. Results

### 3.1. Study Population

**Figure 1:**
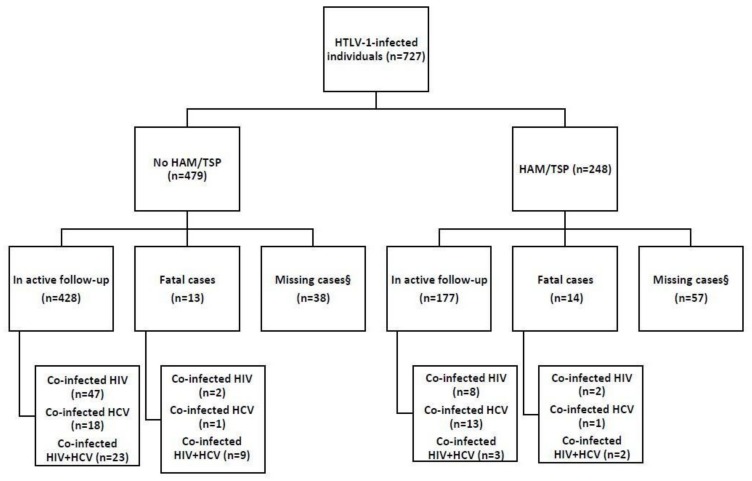
Description of the outcomes of the cohort of people living with Human T-cell Leukemia Virus-1 (HTLV-1) in São Paulo city, Brazil. “HAM/TSP” and “No HAM/TSP” indicate HTLV-1-infected individuals diagnosed with HTLV-1-associated myelopathy/tropical spastic paraperesis (HAM/TSP) at recruitment, or classified as “asymptomatic” during complete neurological examination at recruitment, two of which developed ATLL during follow-up (one fatal case). §Missing cases are those who did not visit the clinic in the last two years and those without complete clinical records.

From July 1997 to December 2018, a total of 727 individuals diagnosed with HTLV-1 infection were recruited and classified as asymptomatic (*n* = 479) or HAM/TSP (*n* = 248) following complete neurological examination [24]. This is an open cohort, with new patients added at a rate of approximately 30–50 per year, also including patients with HIV and/or HCV coinfections. A total of 38 asymptomatic (7.9%) and 57 HAM/TSP cases (23.0%) were excluded from analysis due to loss of follow-up, i.e., no reported visit in the last 24 months. Thus, the total cohort for the current analysis was comprised of 632 subjects (Figure 1).

### 3.2. Clinical Follow-Up

We classified the cohort according to initial diagnosis at recruitment, either “HAM/TSP” or “no HAM/TSP” (Figure 1). During follow-up, two HTLV-1-infected individuals originally diagnosed as “asymptomatic” developed ATLL, one of which died. Although we previously described a subset (42 of 175) of asymptomatics in the cohort which developed an “intermediate syndrome” [26], overlapping with “probable HAM/TSP” according to Castro-Costa criteria, none of these individuals originally diagnosed as “asymptomatic” progressed to clinically definite HAM/TSP and none of them died. Table 1 shows the general characteristics of the deceased individuals from the cohort, as well as the potential risk factors for mortality, including coinfection with HCV and/or HIV. The overall mortality rate was 0.7 per 100 person-years. During a total of 3800 person-years of follow-up (mean follow-up six years), we observed 27 fatal cases (4.3%), of which 13 were asymptomatic (2.9%) and 14 (7.3%) were HAM/TSP patients.

The overall mean age of death was 52.0 years, which was not significantly different between cases with (54.6 years, range 38–78) and without (49.4 years, range 40–68) HAM/TSP diagnosis (Table 1, *p* = 0.23). We observed an overall 0.8:1 female/male ratio among all fatal cases, but an increased 2:1 female ratio for the fatal HAM/TSP cases, although this difference was not statistically significant (Table 1, *p* = 0.17). The main causes of death were infectious, predominated by sepsis and urinary tract infections (UTI) in the whole cohort, as well as among those without HAM/TSP diagnosis. However, the main cause of death among HAM/TSP patients was decubitus pressure ulcers (8/14, 57.1%), which was absent in asymptomatic fatal cases (Table 1, *p* = 0.002). On the other hand, among the HIV/HCV coinfected individuals, the main causes of death (6/13, 46.2%) were sepsis and AIDS -related infections (cryptococcal meningitis and Toxoplasmosis encephalitis), the latter being significantly higher (*p* = 0.033) among fatal cases without HAM/TSP (Table 1). Of the three fatal cases with neoplasia in the cohort, two were linked to oncogenic viruses: one female ATL patient and one male patient (HCV-positive) with hepatocarcinoma.

### 3.3. Survival Analysis and Cox Proportional Hazard Analysis

**Figure 2:**
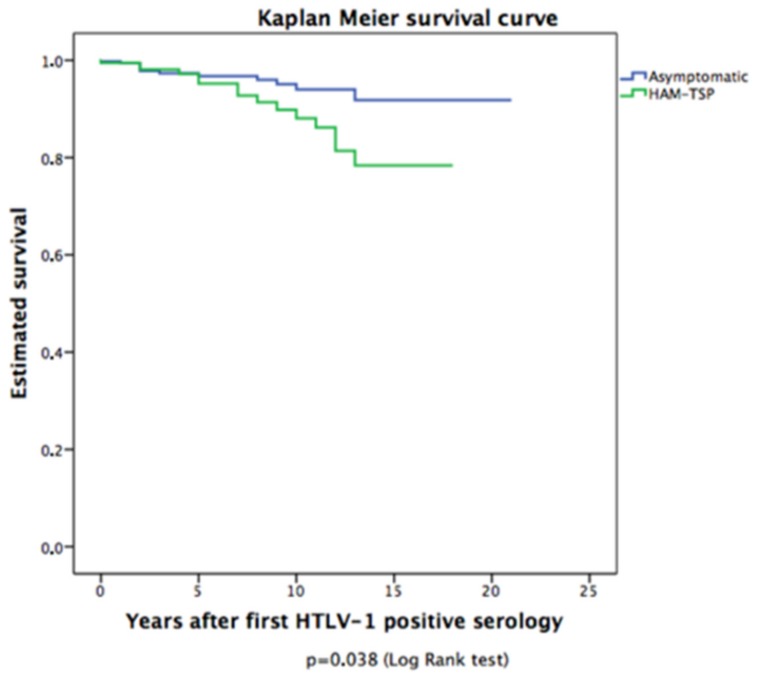
Estimated survival time, according to clinical diagnosis at recruitment (HAM/TSP or asymptomatic).

As evident from Kaplan–Meier curves (Figure 2), the estimated survival time was significantly lower for fatal cases with HAM/TSP diagnosis at recruitment (median survival 16 years) vs. those without HAM/TSP diagnosis (median survival 19 years, *p* = 0.038, log-rank test).

Of note, neither age (HR 1.59, 95% CI 0.73–3.45, *p* = 0.24) nor gender (HR 1.93; 95% CI 0.91–4.13; *p* = 0.089 Wald test) were significantly associated with risk of death by univariate Cox regression analysis (Table 2). However, HAM/TSP diagnosis (HR 2.19; 95% CI 1.02–4.68; *p* = 0.043), HCV infection (HR 5.19; 95% CI 2.44–11.07; *p* < 0.001), HIV infection (HR 5.12; 95% CI 2.39–10.96; *p* < 0.001), and HCV + HIV coinfection (HR 7.48; 95% CI 3.46–16.16; *p* < 0.001) were identified as significant predictors of increased mortality by univariate Cox regression (Table 2).

In multivariate Cox regression models (Table 3), gender was retained but was not found to be an independent predictor (HR 1.16; 5% CI 0.51–2.64; *p* = 0.13). However, both HAM//TSP diagnosis (HR 5.03; 95% CI [1.96–12.91], *p* = 0.001) and coinfection with HIV + HCV (HR 15.08; 95% CI [5.50–41.32]; *p* < 0.001) were identified as independent predictors of the risk of death in our cohort (Table 3).

## 4. Discussion

This open cohort currently has over two decades of continuous follow-up, making it one of the longest running cohorts worldwide to study the clinical outcomes of HTLV-1 infection. During the submission of this manuscript, a systematic review and meta-analysis was published, demonstrating a significantly increased risk (RR 1.57) for mortality in people living with HTLV-1 [20]. Although our cohort recruitment strategy did not include a control group without HTLV-1 infection, our study reveals a young age at death (52 years) and is in agreement with increased mortality in HTLV-1-infected individuals worldwide [20]. Indeed, when comparing with recent Brazilian nation-wide data (www.ibge.gov.br), age- and gender-matched to our cohort, the projected national death rate is 0.56 per 100 person-years, 27% lower than the 0.71 per 100 person-years observed in our cohort.

Several factors might help explain shortened survival among people living with HTLV-1, such as immune senescence and telomere exhaustion triggered during the chronic infection [27]. In addition, lifestyle, socioeconomic conditions, and psychological factors may have an additional influence on these outcomes [28]. Surprisingly, neither age nor gender, the major factors determining survival in the general population as well as most in most cohort studies of people living with HTLV-1 [14,15,16,17,18,19,20,28], significantly predicted mortality in either univariate or multivariate Cox regression models in our cohort. Comparable to other HTLV-1 cohorts and a recent meta-analysis [19,29], neoplasia without viral etiology was infrequent among our fatal cases (one lung carcinoma), while two cases could be linked to oncogenic viruses (one ATLL case and one HCV-positive hepatocarcinoma). However, we identified HAM/TSP diagnosis at recruitment and HIV/HCV coinfection as risk factors for premature death in our cohort.

To our knowledge, this is the first large cohort study describing HAM/TSP as a major and independent risk factor for mortality among people living with HTLV-1. Moreover, the major causes of death among HAM/TSP patients in our Brazilian cohort differ from those described previously in HAM/TSP cohorts in the UK (*n* = 48) and Martinique (*n* = 123), both with a similar long-term follow-up [9,30]. This might be due in part to different definitions of death related to HAM/TSP, namely “pneumonia with respiratory failure, and severe HAM with disseminated inflammation” [30] and “bedridden patients who developed sepsis, pneumonia, nephritis, or pulmonary embolism” [9]. While pneumonia and respiratory disease were not found among fatal cases in our cohort, sepsis occurred at a frequency similar to the Martinique HAM/TSP cohort but did not differ between fatal cases with or without HAM/TSP (Table 1). In contrast, we identified decubitus pressure ulcers, in the absence or presence of (secondary) sepsis, as a major cause of death in HAM/TSP patients. Pressure ulcers and urinary tract infections (UTIs) are frequent, recurrent, and lifelong for patients with neurological impairment [31,32]. A high prevalence of pressure ulcers, especially in those suffering from chronic diseases, has previously been associated to immobility and/or spasticity [32,33,34,35]. Hence, the level of disability might be a possible predictor of mortality, since most (78.6%) of the fatal HAM/TSP cases presented with OMDS over five points (data not shown). Our study provides new insights into the long-term care of people living with HTLV-1, and HAM/TSP patients in particular. Considering the importance of UTI and decubitus ulcers in fatal cases, home visits by a dedicated ‘home care team’, as happened infrequently in this cohort, should be reinforced. Thus, more emphasis should be given to nursing and rehabilitation to prevent UTI and decubitus wounds, especially for HAM/TSP patients with increased disability (OMDS > 5).

In addition, HIV/HCV coinfection was identified as a second, independent predictor of mortality among people living with HTLV-1. Notably, a large proportion of these fatal cases were due to AIDS-related infectious complications (cryptococcal meningitis and toxoplasmosis encephalitis), despite free access to antiretroviral therapy (ART) in Brazil for over two decades. HTLV-1/HIV coinfected individuals in our cohort displayed fourfold higher mortality rates (2.6 per 100 person-years) as compared to recent data from an HIV cohort also located in metropolitan Sao Paulo (0.6 per 100 person-years) [36]. These mortality rates are strikingly similar to a recent publication comparing HTLV-1/HIV-1 coinfected patients (3.0 per 100 person-years) with HIV monoinfected patients (1.3 per 100 person-years) in Salvador-Bahia, Northeast Brazil [13]. However, Brites et al. demonstrated that the decreased survival observed in HTLV-1/HIV-1 coinfected patients was normalized in those with early and successful ART [13].

We acknowledge several limitations in our study, most of which are inherent to the long period of follow-up (>20 years) and the low socio-economic conditions of our population. First, 8% of asymptomatics and 23% of HAM/TSP patients were lost to follow-up, despite our efforts to trace them in the community. However, if we use the 95% CI to extrapolate the 2.9% (95% CI 1.6–4.8%) mortality in individuals without HAM/TSP and the 7.3% (95% CI 4.1–12.0%) mortality in HAM/TSP patients, we can assume another 1–2 and 2–7 fatal cases might have been missed among individuals without and with HAM/TSP, respectively. Although we cannot exclude the possibility that these missing cases might have migrated to another state or region in Brazil, this is highly unlikely for the HAM/TSP cases, considering their limited mobility and low socioeconomic conditions. Second, we do not have data on other important risk factors for mortality, such as smoking, alcohol consumption, and BMI (body mass index). Nonetheless, these factors are more strongly associated to the major causes of death in the general population, namely cardiovascular disease, cancer, and respiratory diseases, which were not among the major causes of death in our cohort. However, a recent study found increased HIV-related mortality associated with lower BMI [37], so this might represent a possible confounding factor in the minor subset (15% of our cohort) HIV coinfected individuals which are over-represented among fatal cases (55%). Third, low income and living outside the large Metropolitan area of São Paulo (the largest city in the Southern hemisphere with an estimated 12 million inhabitants), poses a major hurdle for people living with HTLV-1, and HAM/TSP patients in particular, to comply with yearly visits and hence may influence our results through increased loss of follow-up. The profound economic crisis in Brazil over the last five years might have aggravated this [38], for example, the unemployment rate rose from 7% in 2014 to 13% of the working population in 2017 [39]. Fourth, we do not have data on adherence to antiretroviral therapy (ART) among HIV-coinfected individuals in our cohort. Although all HIV-infected individuals are offered ART as standard-of-care without any cost, as part of Brazil’s public health policy, the high percentage of AIDS-related deaths in our cohort point at low adherence and/or delayed treatment initiation. Collectively, this study reveals the urgent need for public health measures targeted at preventing premature death in people living with HTLV-1. For HAM/TSP patients, both a high loss to follow-up (23%) and UTI or pressure ulcer-related fatal infections indicate the dire need for targeted home interventions. For HTLV-1/HIV coinfected individuals, early access to ART and strategies to increase treatment adherence should be prioritized. In both cases, low socioeconomic status continues as a major hurdle to reduce morbidity and mortality among people living with HTLV-1, reinforcing the need for its recognition as a neglected disease [40].

In conclusion, this study underscores high mortality among people living with HTLV-1 in Sao Paulo, Brazil, and identifies HAM/TSP diagnosis and HIV/HCV coinfection as independent risk factors for death.

## Figures and Tables

**Table 1:** Risk factors for mortality in the HTLV-1 cohort.

Variable	Category	HTLV-1 n (%)	Asymptomatic n (%)	HAM/TSP n (%)	OR	95% CI	*p* Value
**Age**	Mean (SD)	52.2 (10.5)	49.4 (9.2)	54.6 (11.2)			0.23 ^a^
**(years)**	Median	51.5	47	54.5			0.29 ^a^
**Gender**	Male	15 (55.6)	9 (69.2)	6 (42.9)	2.667	0.973–4.430	0.17 ^b^
	Female	12 (44.4)	4 (30.8)	8 (57.1)			
**Coinfected**	HIV	4 (14.8)	2 (15.4)	2 (14.3)	1.543	0.943–2.997	0.39 ^b^
	HCV	4 (14.8)	1 (7.7)	3 (21.4)	2.087	0.936–11.913	0.32 ^b^
	HCV/HIV	11 (40.7)	9 (69.2)	2 (14.3)	2.521	1.141–14.912	0.003 ^b^
**Cause of death ^c^**	Neoplasia	3 (11.1)	2 (15.4)	1 (7.1)	1.625	0.315–8.395	0.60 ^a,^*
	Sepsis	10 (37.0)	6 (46.2)	4 (28.6)	1.471	0.624–3.465	0.44 ^b^
	Decubitus Ulcers	8 (29.6)	-------	8 (57.1)			0.002 ^b^
	Urinary Tract Infection	10 (37.0)	4 (30.8)	7 (50.0)	1.688	0.548–3.196	0.24 ^a,^*
	AIDS-related ^§^	7 (25.9)	6 (46.2)	1 (7.1)	4.550	0.721–3.196	0.033 ^b^

^a^ Mann–Whitney test; ^b^ Fisher test; ^c^ Patients may have more than one cause of death in their medical record; ^§^ Cryptococcal meningitis/Toxoplasmosis Encephalitis.

**Table 2:** Univariate Cox proportional hazard analysis.

Variable	Category	Survivors n (%) (n = 605)	Fatal Cases n (%) (n = 27)	HR	95% CI	*p* Value ^a^
**Age**	≤52 (mean)	51.9 (13.8)	52.2 (10.5)	1.307	0.597–2.859	0.50
	≤53 (median)	53	51.5	1.589	0.731–3.454	0.24
**Gender**	Male	233 (38.5)	15 (55.6)	1.933	0.905–4.130	0.089
	Female	372 (61.5)	12 (44.4)			
**Coinfection**	HCV	67 (11.1)	4 (14.8)	5.194	2.438–11.065	<0.001
	HIV	80 (13.2)	4 (14.8)	5.117	2.388–10.963	<0.001
	HCV/HIV	26 (4.3)	11 (40.7)	7.476	3.458–16.161	<0.001
**Diagnosis**	HAM/TSP	177 (29.3)	14 (51.9)	2.188	1.023–4.678	0.043

^a^ Wald test.

**Table 3:** Multivariate Cox proportional hazard analysis.

Variable	Category	HR	95% CI	*p* Value ^a^
**Coinfection**	HCV/HIV	15.076	5.501–41.318	<0.001
**Diagnosis**	HAM/TSP	5.030	1.959–12.911	0.001
**Gender**	Male	1.161	0.510–2.643	0.13

^a^ Wald test; CI: Confidence interval; HR: Hazard ratio.
